# Multimodal Factor Analysis Reveals Five Robust Phenotypes of Healthy Aging in a Russian Population Cohort

**DOI:** 10.3390/biomedicines14051158

**Published:** 2026-05-20

**Authors:** Lyubov V. Machekhina, Alexandra A. Melnitskaya, Mikhail S. Arbatskiy, Anna V. Permyakova, Alexey V. Churov, Irina D. Strazhesko, Olga N. Tkacheva

**Affiliations:** Russian Clinical Research Center of Gerontology, Pirogov Russian National Research Medical University, Ministry of Healthcare of the Russian Federation, 129226 Moscow, Russiaachurou@yandex.ru (A.V.C.);

**Keywords:** healthy aging, biological age, aging phenotypes, multi-omics integration, MOFA2, multimodal data analysis, biomarkers, personalized medicine, machine learning, Russian cohort

## Abstract

**Background/Objectives:** Population aging necessitates a shift from disease-focused paradigms to a holistic characterization of biological aging processes. While chronological age remains the primary metric, it poorly captures inter-individual variability in physiological resilience and health trajectories. This study aimed to identify robust, multidimensional aging phenotypes independent of chronological age and sex using integrative factor analysis of heterogeneous biomedical data from a Russian cohort—a population underrepresented in aging research. **Methods:** We analyzed data from 1201 conditionally healthy adults (aged 18–99 years) enrolled in the RUSS AGE study. A comprehensive dataset comprising 118 variables across 11 modalities—including biochemical markers, anthropometry, physical function, cognitive-emotional assessments, lifestyle factors, and psychosocial indicators—was integrated using Multi-Omics Factor Analysis v2 (MOFA2). Following the extraction of 16 latent factors and residualization for demographic confounders, consensus clustering was performed to identify distinct aging phenotypes. Phenotype stability was internally recapitulated using gradient-boosting classifiers (XGBoost, CatBoost) in a stratified five-fold cross-validation and on a held-out test set. **Results:** MOFA2 identified 16 stable latent factors, explaining 21.3% of the total variance and capturing coordinated variation across metabolic, inflammatory, cardiovascular, cognitive, and behavioral domains. Consensus clustering revealed five reproducible phenotypes—Anemic (n = 82), Metabolically Subcompensated (n = 99), Metabolically Decompensated (n = 304), Overloaded (n = 302), and Balanced (n = 414)—characterized by distinct multisystem profiles independent of age (*p* > 0.05 after FDR correction) and sex. Supervised classification achieved high discriminative performance (macro F1-score = 0.75, OvR ROC-AUC = 0.93 on the held-out test set), quantifying the internal reconstructability of the phenotype labels from the original feature space rather than external generalization to an independent cohort. **Conclusions:** This study demonstrates the feasibility of data-driven, biologically coherent phenotyping of healthy aging using integrative factor analysis. The identified phenotypes represent stable configurations of physiological, functional, and psychosocial characteristics that transcend chronological age, providing a foundation for the future development of risk-stratification tools, preventive interventions, and biological-age calculators, subject to subsequent validation in longitudinal and independent external cohorts.

## 1. Introduction

Population aging is becoming one of the key global medical and social problems of the 21st century, driving the increased prevalence of chronic non-communicable diseases, multimorbidity, and functional dependence. Contemporary international documents emphasize the necessity of a transition from the paradigm of treating individual diseases to maintaining functional ability and healthy aging throughout the human life cycle [1]. On a molecular level, aging is characterized by a complex interplay of fundamental biological processes. These include genomic instability, epigenetic remodeling, mitochondrial dysfunction, and altered intercellular communication, collectively defined as the hallmarks of aging [2]. Crucially, these conserved mechanisms are considered upstream drivers of the pathophysiological processes that culminate in the majority of age-related diseases. This underscores aging biology itself as a primary etiological factor in chronic multimorbidity, providing a compelling rationale for targeting these hallmarks to delay or prevent age-associated clinical conditions [3].

Significant research efforts are now focused on quantifying the aging process through biomarkers and composite indices of biological age. A critical advancement in this field is the development of multi-marker panels, which have been consistently shown to outperform chronological age as predictors of all-cause mortality, incident age-related disease, and functional decline [4,5,6]. Notably, epigenetic clocks—estimators of biological age derived from DNA methylation patterns—have emerged as powerful tools, demonstrating strong prognostic utility for lifespan and healthspan outcomes [4]. These advances lay the groundwork for operationalizing aging phenotypes, moving beyond single biomarkers to multidimensional constructs.

Critically, age-associated physiological changes do not occur in isolation. Instead, they manifest as a multisystem phenotype, characterized by a network of interconnected alterations across metabolic, inflammatory, cardiorespiratory, neurological, and endocrine axes [3,5,6]. The establishment of large-scale longitudinal cohorts has enabled a paradigm shift towards data-driven phenotyping using cluster analysis methods.

A growing body of research exemplifies this data-driven approach. For instance, Valenzuela et al. (2017) utilized network analysis of integrated physiological and psychological profiles to identify robust clusters with significant prognostic value for mortality [7]. Marron et al. (2019) uncovered substantial heterogeneity in longevity phenotypes within familial cohorts [8], while Halaschek-Wiener et al. (2018) characterized a distinct “Super-Senior” phenotype in individuals who have escaped major age-related diseases [9].

However, these studies are frequently constrained by their reliance on conventional clustering algorithms, such as k-means and hierarchical clustering. These methods possess inherent limitations in managing the analytical challenges posed by modern, complex biomedical datasets—specifically, high dimensionality, data sparsity (missing values), and the need for integrative analysis across disparate data modalities.

Consequently, there is a pressing need for advanced analytical frameworks capable of capturing latent structures within high-dimensional biomedical datasets and integrating information from heterogeneous domains, including clinical chemistry, anthropometry, and behavioral and psychosocial assessments. Methods addressing these challenges are critical for defining coherent aging phenotypes. One such powerful approach is Multi-Omics Factor Analysis v2 (MOFA2), a statistical framework specifically designed for the unsupervised integration of heterogeneous omics and clinical datasets to identify common sources of variation [10]. Simultaneously, novel research is now demonstrating the feasibility of modeling personalized molecular aging trajectories using longitudinal multi-omics profiling [11]. These developments collectively highlight a key frontier in the field: the identification of reproducible and generalizable aging phenotypes that capture fundamental biological processes and translate across diverse populations.

We hypothesized that the multivariate clinical, laboratory, and questionnaire data from our cohort contain latent, integrative axes of variation. These axes, we propose, represent core biological and behavioral processes underpinning inter-individual differences in health status and rates of functional decline. To identify these latent structures in an unbiased manner, we employed Multi-Omics Factor Analysis (MOFA), an unsupervised approach for integrating heterogeneous data. Unlike traditional methods that analyze variables in isolation, MOFA decomposes the complex data matrix into a set of latent factors, each driven by a coordinated pattern of variation across multiple data modalities. We subsequently used these derived factors as a robust foundation for patient stratification via clustering, aiming to discover coherent and clinically interpretable aging phenotypes with enhanced biological validity.

This study presents one of the first applications of integrative probabilistic factor analysis (MOFA2), followed by data-driven phenotyping of a multidimensional biomedical dataset from a Russian population. The majority of existing research on aging phenotypes and biomarker integration relies on data from large, well-established Western cohorts, such as the UK Biobank [12] and the Estonian Biobank [13]. Our work addresses a significant geographical gap in the literature by identifying aging profiles within a distinct population context.

While these international biobanks are invaluable resources, their findings may not be directly generalizable to the Russian population. Geographic and population-specific factors-including genetic background, environmental exposures, and socioeconomic conditions—can significantly influence biomarker distributions, risk factor profiles, and the manifestation of aging phenotypes. Although several notable Russian cohorts exist—e.g., ESSE-RF [14], HAPIEE [15], EVKALIPT [16]—their primary focus has been on discrete clinical endpoints, such as specific diseases, cardiovascular outcomes, and cognitive decline, rather than on integrative, data-driven phenotyping of the aging process itself.

In contrast to these disease-focused cohorts, our study is specifically designed to phenotypically characterize healthy aging in a predominantly healthy adult sample. This approach allows us to delineate multi-domain health profiles that are not confounded by major clinical diagnoses, thereby capturing the inherent variability of normative aging. Given the high dimensionality and heterogeneity of such data, and the absence of established phenotypic benchmarks for the Russian population, there is a clear need for analytical methods that can discover latent structure without relying on predefined clinical categories. To meet this need, we employed the probabilistic factorization framework MOFA2. This method is suited to integrate heterogeneous data types—laboratory markers, anthropometry, functional assessments, and psychosocial scales—and to reconstruct the latent axes of covariation that form the basis of phenotypic diversity in healthy aging.

## 2. Materials and Methods

### 2.1. Subjects and Sample

The present analysis was performed within the RUSS AGE study, a single-center, cross-sectional investigation of aging conducted in Russia. The detailed study protocol has been published previously [17]. All statistical and bioinformatic analyses were executed using Python (version 3.10). Primary data manipulation and numerical computations were carried out with the pandas (v2.1.4) and numpy (v1.26.0) libraries, respectively. For the core dimensionality reduction and data integration, we employed the MOFA2 (mofapy2 v0.7.3) framework via its dedicated Python implementation. Subsequent analyses, including clustering and model evaluation, utilized functionalities from the scikit-learn library (v1.7.2).

The analytical cohort included 1201 conditionally healthy volunteers aged 18 to 99 years (median [IQR]: 46 [33–64] years; mean ± SD: 49.3 ± 19.6 years). Participants were predominantly female (74%, n = 889), with 26% (n = 312) male. The classification as “conditionally healthy” was based on self-reported data from structured questionnaires and voluntarily provided personal medical documentation. Primary exclusion criteria, as detailed in the original protocol [17], included: (1) significant sensory or cognitive impairments, (2) acute or life-threatening medical conditions, and (3) a prior diagnosis of major chronic disorders spanning cardiovascular, oncological, endocrine, and autoimmune diseases, among others.

For each participant, a comprehensive dataset was collected, comprising 118 variables distributed across thematic modalities reflecting various domains of biological and functional status: sex, age, blood pressure, body mass index, education, employment, marital status, having children, having pets, religious affiliation, income level, smoking, alcohol consumption, physical activity of various intensities, medical and medication history, self-rated general health, cognitive-emotional indicators, sleep parameters, dietary habits, laboratory parameters—biochemical, hormonal, and inflammatory markers.

The study was conducted at the Russian Gerontology Clinical Research Center of the Pirogov Russian National Research Medical University (Moscow) between 2022 and 2024.

The distribution of participants by 10-year age strata was: 18–29 years—220 (18.3%); 30–39—244 (20.3%); 40–49—191 (15.9%); 50–59—180 (15.0%); 60–69—139 (11.6%); 70–79—115 (9.6%); 80–89—79 (6.6%); and 90+—33 (2.7%). The age-by-sex composition (Supplementary Figure S1) reflects the typical demographic skew towards women in older age groups (female median 50 y [IQR 36–67]; male median 36 y [IQR 29–53]).

Each participant underwent a single-visit structured assessment comprising (i) self-administered and interview-based questionnaires covering demographics, lifestyle, dietary habits, sleep, cognitive–emotional state and self-rated health/subjective-age perception; (ii) anthropometric measurements (height, weight, body-mass index, waist/arm/calf circumference, blood pressure, heart rate); (iii) physical-function tests (hand-grip dynamometry of both hands, timed chair-stand without hand support, Trail-Making Test parts A and B, Symbol-Digit Modalities Test, semi-tandem stand); and (iv) venous blood sampling after a 10–12-hour overnight fast, with specimen processing and storage at −80 °C in the RUSS-AGE biobank for biochemical, hormonal and inflammatory biomarker assays.

After preprocessing and quality control, 118 variables were retained for MOFA2 integration, distributed across 11 thematic modalities (see Supplementary Table S1 for the complete variable dictionary). A dedicated English-language cohort description manuscript is in preparation.

### 2.2. Data Preprocessing Before Model Input

To prepare the heterogeneous dataset for multimodal integration, a standardized preprocessing pipeline was implemented. All continuous variables were centered and scaled to unit variance (z-score normalization) within each column. Categorical variables were converted into binary variables using one-hot encoding. In accordance with the MOFA2 framework, which allows for different likelihood models per data modality, processed continuous and scale variables were treated as following a Gaussian distribution, while the newly created binary variables were modeled using a Bernoulli distribution.

For scale data (questionnaires), items with positive directionality were also reverse-coded to ensure that higher values across all variables corresponded to greater symptom or sign severity, facilitating clinical interpretation of the factors. Special consideration was given to age-related measures. To model the subjective perception of aging independently from chronological age, we calculated derived “age gap” variables by subtracting an individual’s chronological age from their self-reported subjective age estimates. This approach allowed the integration of subjective age perception as a distinct construct, avoiding collinearity with the core demographic covariate.

No imputation of missing values was performed, and samples were not excluded due to missing data. The analysis was conducted using all available data, allowing the retention of the full cohort and maximizing the use of available information, a feature inherently supported by the MOFA2 framework. Following preprocessing, each data modality was stored as a separate, standardized matrix. The final integrated dataset for MOFA2 training comprised 11 modalities: 9 with continuous data (modeled with a Gaussian likelihood) and 2 with binary data (modeled with a Bernoulli likelihood). The number of variables per modality ranged from 5 to 40.

A per-modality breakdown of the preprocessing steps—including the likelihood family (Gaussian vs. Bernoulli) and explicit examples of one-hot variants generated from categorical questionnaire items—is provided in Supplementary Table S1 (sheet “Modality_Summary”). Nine of the eleven modalities use a Gaussian likelihood on standardized continuous variables; the Social view (15 binary features) and the Behavioral-risk (binary) view (6 binary features) use a Bernoulli likelihood on one-hot-encoded categorical indicators. The final MOFA2 input, therefore, comprised 118 features (97 Gaussian + 21 Bernoulli).

### 2.3. MOFA2 Model Training and Configuration

The preprocessed data modalities were integrated using the Multi-Omics Factor Analysis 2 (MOFA2) framework, a Bayesian factor model designed for the unsupervised integration of heterogeneous data types via probabilistic matrix factorization [10]. To identify the optimal latent dimensionality, models were trained with varying numbers of latent factors (K = 8, 10, 12, 14, 16). For each value of K, the model was run three times with different random initializations (seeds: 17, 42, 2025) to assess its stability and reproducibility. All runs used the robust “medium” convergence setting and ran for 2500 iterations.

Model stability was ensured by applying feature scaling per modality (scale_views = True) and Automatic Relevance Determination (ARD) priors, which automatically regulate the contribution weight of each data modality to the latent factors. The final model, selected based on maximum total explained variance across all views, contained K = 16 latent factors (EV_total ≈ 0.213). All downstream analyses—including factor interpretation, phenotypic clustering, and validation—were conducted using this optimal configuration.

No orthogonality constraint is imposed on the latent factors. MOFA2 uses independent Gaussian priors on the factor-score matrix Z combined with Automatic Relevance Determination (ARD) priors on the loadings [10,18]; any low empirical collinearity between factors is therefore an a posteriori property of the fitted model rather than a methodological constraint (see Section 3.2 for the empirical factor-correlation values).

### 2.4. Data Processing for Factor Interpretation

To facilitate clinically meaningful interpretation of the latent factors, two key preparatory steps were undertaken: statistical adjustment for demographic confounders and the creation of a standardized annotation resource.

Residualization of Latent Factors: The influence of core demographic variables was removed from each factor’s scores via multiple linear regression. For each of the 16 factors, a model was fitted with the factor score as the outcome and predictors for chronological age (including a quadratic term) and sex. The resulting residuals were used in subsequent analyses.

Creation of a Clinical Variable Dictionary: A unified dictionary was compiled as an Excel table to standardize the interpretation of factor loadings and to aid in visualization. This dictionary lists all input variables.

The residuals derived from the regression models described above were treated as demographically adjusted (residualized) factor scores. These adjusted factor values were used in all subsequent analyses. Subsequently, a dedicated clinical variable dictionary was constructed. This dictionary mapped the 118 analyzed variables from their internal model identifiers to their full clinical descriptions, serving as an essential reference for interpreting factor loadings and defining clinically coherent phenotypes based on the latent dimensions.

Throughout this work, the term “factor score” refers to the posterior mean of the latent-factor matrix Z (shape N × K = 1201 × 16), extracted directly from the trained MOFA2 model. No additional post-processing (rotation, rescaling, orthogonalisation) was applied prior to demographic residualization and downstream analyses. The complete matrix of factor loadings across all 118 variables and 16 latent factors, together with ranked top-15 positive/negative loadings per factor and a semantic-theme summary, is provided in Supplementary Table S2. The clinical variable dictionary is provided in Supplementary Table S1. A quantitative before/after comparison of factor–age correlations and factor–sex effects is shown in Supplementary Figure S2.

### 2.5. Phenotypic Clustering and Classification

Clustering was performed on the residualized values of the 16 MOFA2 latent factors, reflecting the main biological axes of data variability. The k-means algorithm [19] was used in bootstrapping mode followed by consensus clustering. The optimal number of clusters was determined based on standard stability metrics, including the Proportion of Ambiguous Clusters (PAC) [20] and the Adjusted Rand Index (ARI) [21], in combination with the clinical interpretability of the solution. The final partition into five phenotypes (K = 5) was formed from the consensus matrix using hierarchical aggregation and clinical interpretation.

**Figure 1:**
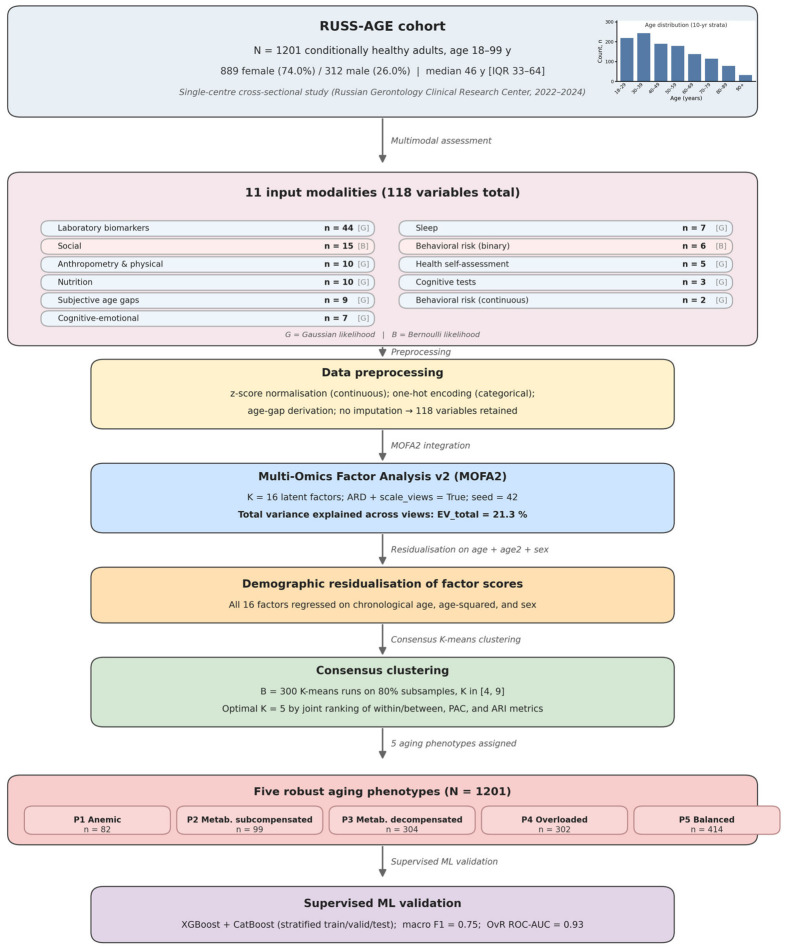
Detailed analytical pipeline of the RUSS-AGE phenotyping analysis. The cohort block shows sample size, sex composition, and age distribution (inset histogram, 10-year strata). The eleven input modalities are listed with their per-modality variable counts and likelihood family (Gaussian or Bernoulli). Subsequent blocks describe data preprocessing (yielding 118 retained variables), MOFA2 integration (K = 16 latent factors, total variance explained 21.3%), demographic residualization, consensus K-means clustering (optimal K = 5 by joint ranking of within/between/PAC/ARI metrics), the resulting five aging phenotypes with their sample sizes, and the downstream XGBoost/CatBoost supervised validation (macro F1 ≈ 0.75, One-vs-Rest ROC-AUC ≈ 0.93). G = Gaussian likelihood; B = Bernoulli likelihood.

The key laboratory markers distinguishing the phenotypes were determined by the magnitude of standardized effects (Standardized Mean Difference, Cliff’s δ) in comparison with the rest of the sample. For a preliminary check of the reproducibility of the phenotypic structure and an assessment of its potential for predictive tasks, multiclass models based on gradient boosting (XGBoost v2.0.3 [22], CatBoost v1.2.2 [23]) were built. The models were trained in the stratified five-fold cross-validation mode. Detailed results of the machine-learning validation are presented in Section 3.5; the overall analytical pipeline is shown in Figure 1.

To clarify the exact sequence of steps applied in the supervised machine-learning validation, we provide the following step-by-step description. The validation is of a “reconstructability” type: it tests whether unsupervised phenotype labels can be recovered from the same biomedical features that originally generated them, using a standard supervised classifier.

Step 1 (fit once, on the full cohort). The MOFA2 model (K = 16), the demographic residualization, and the consensus K-means clustering (B = 300 bootstraps, K = 5) are fitted once on the full cohort (N = 1201). At the end of Step 1, each participant has a definitive phenotype label in {P1, P2, P3, P4, P5}.

Step 2 (data split). The matrix of original pre-MOFA biomedical variables X and the phenotype vector y are split into training (70%, N = 841), validation (15%, N = 180), and test (15%, N = 180) sets, stratified by y (RANDOM_STATE = 42).

Step 3 (cross-validation within the training set). StratifiedKFold (scikit-learn v1.7.2) with five folds is used on the training set. In each fold, an XGBoost multiclass classifier is fitted on 80% of the training set and evaluated on the remaining 20%; macro F1 and one-vs-rest ROC-AUC are reported as mean ± SD across the five folds. A CatBoost model is trained in parallel on the same folds for algorithm-agnostic comparison.

Step 4 (held-out test). The XGBoost model from Step 3 (refit on the full 70% training set with best-iteration selection from the validation set) is then evaluated once on the held-out 15% test set.

Step 5 (reporting). The final reporting step produces one-vs-rest ROC curves on the 15% held-out test set with per-phenotype AUC values in the legend (presented in the Results, Section 3.5). The classifier’s F1 and AUC values on the five-fold cross-validation and held-out test agree within 0.002 and 0.001, respectively.

Importantly, MOFA2 and clustering are not refit per fold. This choice is deliberate: per-fold refitting would produce a different latent structure in each fold, breaking the correspondence between cross-fold phenotype labels. The reported ROC-AUC values, therefore, quantify the reconstructability of the labels from the same feature space, not out-of-sample generalization to an independent cohort; this distinction is further discussed in Section 4.5 (Limitations).

## 3. Results

### 3.1. MOFA2 Model Training and Factor Extraction

Integrative factor modeling of the 11 input modalities using MOFA2 revealed a robust latent structure that captures the primary axes of variation in participants’ biological and psychosocial profiles. The model with optimal dimensionality K = 16 latent factors explained approximately 0.213 of the total variance (EV_total) across all data views. This solution demonstrated excellent reproducibility, as evidenced by a perfect correlation (mean Procrustes correlation = 1.0) between factor weights across three independent model runs initialized with different random seeds. Given its optimal balance between explained variance and stability, the 16-factor model was selected for all subsequent downstream analyses.

### 3.2. Description of Latent Factors

The sixteen latent factors identified by the MOFA2 model collectively captured the multivariate structure of the data. Their contributions to the total explained variance followed a moderate gradient: factors 1–6 were the most influential, each explaining approximately 7–8% of the variance. Factors 7 through 12 accounted for a slightly lower proportion (5–6% each), while factors 13 through 16 explained 4–5% each. To interpret the resulting phenotypic clusters, we calculated the mean factor score profiles (Z-scores) for each cluster. These profiles were based on residualized factor scores, adjusted for linear and quadratic age and sex to remove the influence of these demographic confounders.

Each latent factor was characterized by a distinct pattern of positive and negative deviations in its Z-score profile, representing a coordinated pattern of variation across metabolic, inflammatory, endocrine, cognitive, and behavioral domains. The comparative analysis of these profiles revealed that none of the phenotypic clusters could be attributed to a single dominant factor. Instead, each phenotype emerged as a specific configuration of simultaneous, correlated shifts across multiple physiological systems, highlighting the multisystem nature of the aging process captured by our model.

Demographic analysis revealed that Factor 1 exhibited the strongest association with chronological age, demonstrating a very strong inverse correlation (r = −0.89). Notably, although chronological age was excluded as an input variable during model training, this factor captured a robust, coordinated gradient of age-related variation across multiple data modalities.

**Figure 2:**
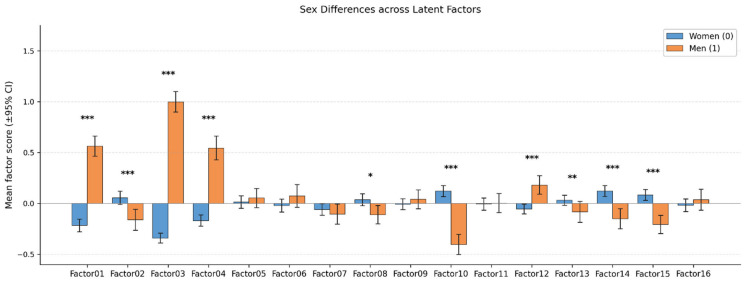
Sex-related differences in MOFA2-derived latent factor scores (raw, non-residualized). Bars show mean factor scores; the vertical whiskers (error bars) on each bar represent the 95% confidence interval of the mean. Female (n = 889) and male (n = 312) participants are shown separately. Because factor scores are mean-centered across the full sample, the weighted group means are algebraically constrained to sum to zero—the smaller minority group (26% male), therefore, appears shifted symmetrically proportionally in the opposite direction whenever the female mean deviates from zero. The direction of deviation for each factor is biologically interpretable from the top positive and negative loadings (Supplementary Table S2). Statistical significance of inter-sex differences was assessed using an independent-sample *t*-test (*** *p* < 0.001; ** *p* < 0.01; * *p* < 0.05). These raw sex effects are eliminated by the residualization step applied prior to clustering (Methods Section 2.4; see Supplementary Figure S2 for a quantitative before/after comparison).

Several other factors showed statistically significant, although weaker, correlations with age: Factor 5 (r = 0.28), Factor 14 (r = 0.14), Factor 4 (r = 0.11), and Factor 15 (r = −0.07). The remaining factors displayed no significant age-related associations (absolute r ≤ 0.052). Analysis of sex differences in the latent factor scores (Figure 2) indicated that statistically significant sex effects were confined to a specific subset of factors.

Significant sex-related disparities were most prominent for Factors 1, 3, and 4, with males exhibiting significantly higher factor scores compared to females (*p* < 0.001). Moderate but statistically significant differences (*p*-values between approximately 0.01 and 0.05) were observed for Factors 2, 8, 10, 12, 13, 14, and 15. An interpretable pattern emerged from these differences: higher scores in males were typically associated with factors loaded on physiological and metabolic traits, whereas higher scores in females were linked to factors capturing aspects of emotional and social regulation.

The majority of latent factors (specifically 5, 6, 7, 9, 11, and 16) showed no statistically significant sex-based differences (*p* ≥ 0.05). Overall, the influence of sex within the derived factor space was moderate and largely confined to physiological and metabolic domains, while cognitive and psychoemotional dimensions remained comparable between genders.

Pairwise Pearson correlations between the 16 posterior factor scores were computed directly from the trained MOFA2 model. All 120 inter-factor correlations fell within |r| < 0.16 (median |r| = 0.03; 95th percentile |r| = 0.10; maximum |r| = 0.157). Because MOFA2 does not impose orthogonality (Section 2.3), this near-orthogonal structure is an empirical consequence of the ARD priors and the independent Gaussian priors on Z [10,18] rather than an algorithmic constraint. It nonetheless indicates that each factor captures a distinct, non-redundant axis of variation in the integrated dataset.

Factor 1 captured a dominant integrative age-metabolic gradient. It was strongly loaded on variables related to glucose and lipid metabolism, muscle strength, and self-rated health, exhibiting a very strong negative correlation with chronological age (r = −0.897).

Factors 2 and 3 were primarily associated with psychological and cognitive profiles. Their high-loading variables pertained to perceived stress levels and performance in domains such as attention, information processing speed, and psychomotor coordination.

Factor 4 captured variance primarily associated with the level of physical functioning, including metrics of activity intensity, muscle strength, and balance.

Factors 5 and 6 reflected variance predominantly in systemic physiological circuits. Factor 5 was characterized by lipid and cardiometabolic parameters (e.g., blood pressure, blood lipids), while Factor 6 was associated with hepatic and renal functional markers (e.g., creatinine, liver enzymes).

Factors 7 and 8 captured variance associated with lifestyle patterns, social engagement, and subjective well-being.

Factor 9 was related to sleep quality and circadian regulation.

Factors 10 and 15 reflected aspects of behavioral risk, aggregating variables related to habits, sleep disturbances, and biomarkers of stress.

Factor 11 was associated with endocrine and mineral metabolism, primarily loaded on variables such as vitamin D and alkaline phosphatase.

Factor 12 integrated markers of cardiovascular and autonomic function, including blood pressure, electrolyte balance, and indicators of autonomic tone, suggesting a coordinated axis of cardiovascular-endocrine regulation.

Factors 13 and 14 captured dimensions related to affective state and dietary behavior patterns.

Factor 16 reflected a pattern of neuroendocrine adaptation, characterized by loadings on cortisol, other stress-related biomarkers, and sleep parameters.

### 3.3. Phenotypic Clustering Based on Latent Factors

**Figure 3:**
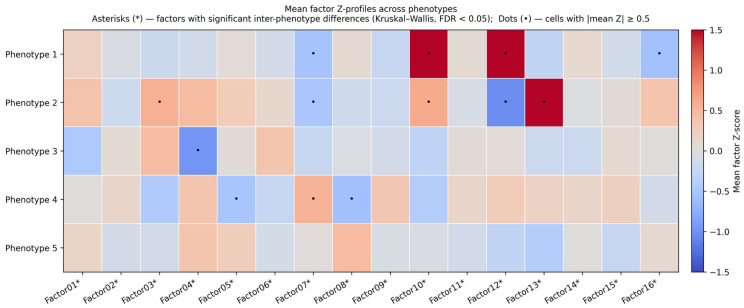
Mean Z-score profiles of the 16 residualized MOFA2 latent factors across the five phenotypic clusters identified by consensus K-means. Residualization was performed by regressing each factor on chronological age, age squared, and sex (Methods Section 2.4); the resulting residuals are independent of the demographic covariates (max |Spearman ρ with age| < 0.05 and max |Cohen’s d by sex| ≈ 0 across all 16 factors; see Supplementary Figure S2 for a quantitative before/after comparison). Cell color encodes the mean residualized factor Z-score per phenotype (blue = below cohort mean, red = above). Asterisks (*) next to factor labels denote factors with statistically significant inter-phenotype differ-ences (Kruskal–Wallis test, FDR-adjusted *p*-value < 0.05). Dots (•) inside cells mark phenotype-factor combinations where the absolute mean Z-score reaches or exceeds 0.5 SD — i.e., a notable (moderate-to-large) deviation of that phenotype from the cohort centre on that factor.

To identify robust biological subgroups, we performed consensus clustering on a matrix of residualized MOFA2 factor scores, which were adjusted for age, quadratic age, and sex. We evaluated clustering solutions across a range of potential cluster numbers (K = 4–9). Model selection was guided by cluster stability metrics: the PAC, where lower values indicate greater stability, and the ARI, which measures the consistency of partitions across algorithm iterations (higher values preferred). K = 5 was identified as the jointly optimal partition by sum-of-ranks across all four metrics (within-cluster compactness = 0.86, between-cluster separation = 0.04, PAC = 0.28, ARI_bootstrap = 0.69 over B = 300 resamples). The neighboring solution K = 6 exhibited a markedly lower ARI (0.50) with comparable within/between values, indicating that the sixth cluster did not stabilize across bootstrap resamples and that the partition became less reproducible beyond K = 5. Solutions at K ≥ 7 showed further ARI decay (0.45, 0.40, 0.36 for K = 7, 8, 9, respectively), consistent with over-partitioning, while K = 4 collapsed the Anemic phenotype into a larger group (ARI = 0.58). The full cluster-K grid, MOFA-K sensitivity, and factor-subset sensitivity are reported in Supplementary Table S3. The mean residualized factor-score profiles of the resulting five phenotypes are shown in Figure 3.

The identified clusters represent five robust trajectories of alteration across biological and functional domains, supporting their definition as distinct, clinically meaningful phenotypes of healthy aging. To verify that these phenotypes were not driven by demographic confounders, we repeated the clustering analysis using the original, non-residualized factor scores. The resulting cluster structure remained largely unchanged, confirming that the identified phenotypes are independent of age and sex.

Analysis of the age distribution across the five phenotypes revealed moderate inter-group differences and no strong age gradient. The median age of participants varied between phenotypes, ranging from 38 to 52 years. Specifically, Phenotype 2 comprised the youngest individuals (median age 38 years), whereas Phenotype 3 included the oldest participants (median age 52 years).

The wide interquartile ranges observed within each phenotype indicate substantial internal age heterogeneity, confirming that chronological age was not the primary driver of the cluster separation. Although a Kruskal–Wallis test detected nominally significant inter-phenotype differences in age (*p* = 0.001), this significance did not survive correction for multiple testing (e.g., using FDR adjustment). This further underscores that the phenotypes were relatively balanced with respect to age.

The distribution of sex across the phenotypes did not reveal any significant disproportions. A moderate predominance of female participants was maintained across all groups (ranging from 54% to 87%), with no systematic gradient. While Phenotype 2 contained the highest proportion of males (46%) and Phenotype 1 the lowest (13%), these differences were not statistically significant, indicating no strong association between the derived phenotypes and biological sex.

To evaluate the phenotypic profiles relative to the overall sample, we calculated the position of each cluster centroid within the 16-dimensional MOFA2 factor space. The methodology corresponds to the approach by Valenzuela et al. [7], where the distance from the global center reflects the degree of biological vulnerability or well-being. In our analysis, Phenotype 5 was located closest to the global origin (distance = 0.903), suggesting it represents the most normative or physiologically balanced profile. Phenotypes 3 and 4 occupied intermediate positions. In contrast, Phenotypes 2 (distance = 2.491) and 1 (distance = 2.844) were the most distant, indicating the most distinct and potentially adverse biological configurations.

**Figure 4:**
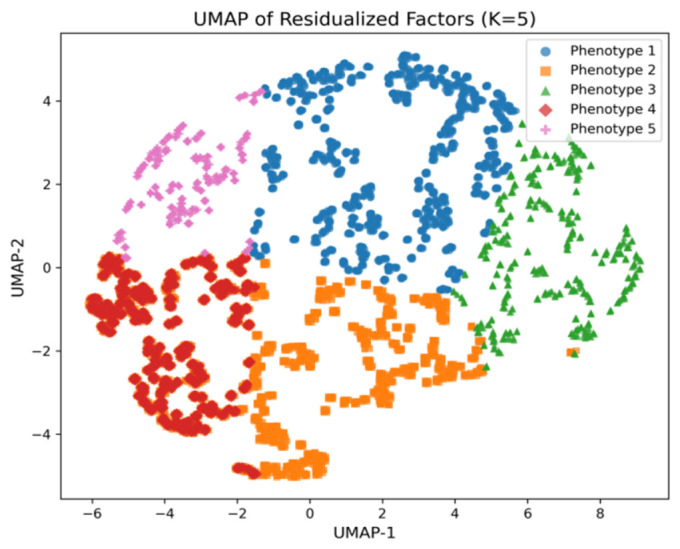
Uniform Manifold Approximation and Projection (UMAP) visualization of the five identified phenotypic clusters (K = 5), based on the residualized MOFA2 latent factor scores. The plot illustrates the spatial separation and distribution of clusters corresponding to healthy and adverse aging phenotypes.

PCA projection confirms these differences: Phenotype 5 is located at the center of the data cloud, while Phenotypes 1 and 2 are shifted to opposite sides of the factor space. Informed by the dominant biochemical and functional patterns characterizing each cluster, we assigned the following descriptive labels: (1) Anemic, (2) Metabolically Subcompensated, (3) Metabolically Decompensated, (4) Overloaded, and (5) Balanced (Figure 4). A detailed clinical characterization of each phenotype, including associated behavioral, psychoemotional, and laboratory profiles, is provided in Section 3.4 and in Supplementary Table S4.

### 3.4. Phenotype Characterization

Phenotype 1 (Anemic)—n = 82 (6.8%). Predominantly young women (87%; median age 42 years [IQR 33–49]). The defining laboratory pattern is a latent iron-deficient anemia with mild inflammatory overlay: markedly reduced MCV, ferritin, hemoglobin, and haematocrit, together with elevated RDW, ESR, platelets, and IL-6. Secondary features include slightly elevated leptin and insulin with normal-to-low waist circumference, consistent with an adaptive hypermetabolic response to energy deficit. Behaviourally, participants report lower physical activity, stress-related eating (frequent sweets and desserts, infrequent vegetables and dairy), and mild dissatisfaction with sleep without frank insomnia. The pattern corresponds clinically to a chronic-fatigue/anemic phenotype vulnerable to immunometabolic decline if untreated.

Phenotype 2 (Metabolically subcompensated)—n = 99 (8.2%). The youngest phenotype (median age 37 y [28–58]) and the most sex-balanced (55% female). It combines elevated glucose, HbA1c, fructosamine, insulin, C-peptide, ferritin, and alkaline phosphatase with high grip strength and larger arm, waist, and calf circumferences—an “athletically hyperglycaemic” signature typical of an anabolic phenotype in the early metabolic-syndrome spectrum, yet without overt obesity or depressive symptoms. Lifestyle includes frequent fast-food and sweet-snack intake, but active employment and social participation. Sleep is largely preserved.

Phenotype 3 (Metabolically decompensated)—n = 304 (25.3%). The largest middle-aged phenotype (median age 52 y [33–66]; 73% female). The biochemical picture is a full inflammatory-metabolic syndrome with abdominal obesity: high BMI, waist and arm circumferences, elevated insulin, C-peptide, hs-CRP, ferritin, uric acid, ALP, and glucose, together with early systolic/diastolic hypertension and elevated leukocyte/neutrophil/platelet/ESR. Subclinical endothelial and vascular-aging biomarkers (homocysteine, MMP-9, cystatin C) are also elevated. Behaviourally, the phenotype is characterized by low physical activity, poor diet quality, fragmented sleep, and daytime fatigue. Educational attainment and employment are modestly lower. Clinically, this is an insulin-resistant/inflammatory profile in early metabolic syndrome.

Phenotype 4 (Overloaded)—n = 302 (25.1%). Working-age adults with a notable dominance of women (77%; median age 49 y [34–66]). Paradoxically, the biochemical profile is among the healthiest in the cohort: elevated 25(OH) vitamin D, albumin, HDL, MCV, hemoglobin, SHBG, IGF-1, and Klotho, with reduced insulin, C-peptide, and ALP—a “metabolically compensated” fingerprint. Cognitive test performance is above the sample mean, and self-rated youthfulness is high. However, participants uniformly report subjectively poor sleep quality alongside high daytime demand, yielding a “high-functioning but stress-sensitive” or “overloaded-healthy” clinical profile. Diet is more Mediterranean-style, and sedentary time is lower than in Phenotype 3.

Phenotype 5 (Balanced)—n = 414 (34.5%). The largest and centroid-closest phenotype (median age 45 y [33–63]; 75% female). Its name reflects its central position in the 16-factor space rather than a uniformly pristine biochemistry: mild astheno-inflammatory markers are observed (elevated creatinine, cystatin C, urea, NT-proBNP, homocysteine, and IL-6; slightly reduced albumin), and sleep quality is the poorest in the cohort on subjective measures (fragmented sleep, early awakening, low satisfaction). Nonetheless, cognitive performance and grip strength remain within the sample average. Thus, the phenotype represents an adaptive-compensation state in which mild multisystem strain is balanced by preserved function. Clinically, it is an astheno-inflammatory/under-recovered profile with conserved daily activity.

A complete per-phenotype characterization—including sample composition, top distinguishing biomarkers with Cliff’s delta effect sizes and behavioral/social/clinical profiles—is provided in Supplementary Table S4.

### 3.5. Preliminary Validation of Phenotypes

To determine whether the derived aging phenotypes could be reliably predicted from raw clinical, laboratory, and survey variables, we trained a supervised multiclass classifier using the XGBoost algorithm. Model performance was rigorously assessed via a stratified five-fold cross-validation. The classifier achieved high predictive accuracy, with a macro-averaged F1-score of 0.749 ± 0.023 and a One-vs.-Rest (OvR) ROC-AUC of 0.930 ± 0.014. Performance on a held-out independent test set was consistent (accuracy = 0.748, OvR ROC-AUC = 0.931), confirming the internal recapitulability of the phenotype labels from the original feature space. As discussed in Section 2.5, this metric quantifies internal reconstructability rather than external generalization to an independent cohort. Therefore, the supervised step is an algorithm-agnostic consistency check of the unsupervised partition rather than an out-of-sample prediction test.

**Figure 5:**
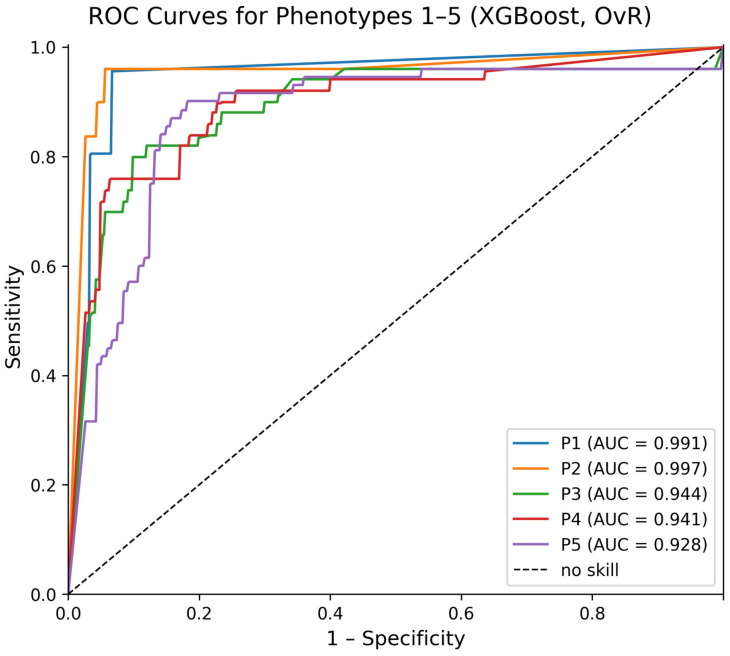
One-vs.-rest (OvR) receiver operating characteristic curves for the five aging phenotypes, computed on the held-out 15% test set (N = 180; see Methods Section 2.5 for the complete validation pipeline). Phenotype labels were obtained from the unsupervised MOFA + consensus-clustering pipeline, which was fitted once on the full cohort. The XGBoost classifier learns to recover these labels from the original biomedical variables. AUC values are given in the legend, where Px denotes phenotype x (P1 = Anemic; P2 = Metabolically Subcompensated; P3 = Metabolically Decompensated; P4 = Overloaded; P5 = Balanced; see Section 3.4 for the full phenotype definitions). The dashed diagonal line represents the performance of a no-skill classifier (AUC = 0.5).

Analysis of classification errors revealed the highest degree of confusion between Phenotypes 3 and 4, aligning with the partial overlap in their metabolic and hormonal profiles. In contrast, Phenotypes 1 and 5 were the most distinguishable, with individual F1-scores of 0.81 and 0.85, respectively. The one-vs-rest (OvR) area under the receiver operating characteristic curve (AUC) for each phenotype further confirmed the model’s high discriminative power: Phenotype 1 (AUC = 0.991), Phenotype 2 (AUC = 0.997), Phenotype 3 (AUC = 0.944), Phenotype 4 (AUC = 0.941), and Phenotype 5 (AUC = 0.928) (Figure 5).

To ensure our findings were not dependent on a single algorithm, a CatBoost model was trained, showing comparable metrics (F1_macro ≈ 0.75, ROC-AUC ≈ 0.93) and nearly identical feature ranking. These data indicate that the phenotypes represent stable classes distinguishable by the original biomedical, functional, and questionnaire characteristics; detailed analysis of feature importance and partial dependencies will be presented in a separate article.

## 4. Discussion

### 4.1. Validation and Interpretability of MOFA2 Latent Factors

Following MOFA model training, we sought to evaluate whether the derived latent factors captured coherent systemic biology rather than statistical noise. We analyzed the distribution of explained variance (EV) across both the latent factors and modalities. The overall EV profile indicated that the model successfully avoided extracting noise-driven components: each of the 16 latent components makes a significant contribution, and their relative evenness indicates the capture of multiple independent biological axes.

We quantitatively evaluated the factorization quality by examining the total variance explained (EV_total) across all data modalities, which summarizes the overall proportion of data variability captured by the latent factors. Our model achieved an EV_total of 21%. This value falls within the typical range (≈18–30%) reported for MOFA/MOFA+ models in foundational methodological studies [10], confirming the successful and appropriate integration of our heterogeneous data views.

Subsequent methodological reviews of the MOFA+ framework indicate that a total explained variance in the range of 15–30% is typical when integrating highly heterogeneous data types. This range reflects the inherent challenge of capturing shared variation across distinct biological layers; this is considered normative for probabilistic factorization models, signifying the successful identification of robust inter-modal structures rather than a methodological limitation [18]. This is consistent with applied studies; for example, Canzler et al. reported that MOFA captured up to 30% of the variance in proteomic and metabolomic datasets within an integrated multi-omics analysis [24].

Subsequently, we sought to interpret the latent factors as independent biological axes. A critical methodological decision was to exclude chronological age as an explicit variable from all input modalities. This approach is supported by the established literature indicating that chronological age acts as a global driver of inter-individual variance across a wide array of biomarkers. Its inclusion in factor models often results in a single dominant factor capturing this age-related variation, thereby obscuring the more subtle, system-specific biological axes of interest [2,3].

Excluding chronological age enabled the model to disentangle genuine biological and behavioral axes of variation from demographic gradients. Post hoc analysis confirmed that, despite its exclusion, five of the sixteen latent factors still exhibited a significant statistical association with age. The remaining factors captured age-independent sources of interpersonal variance, representing distinct patterns in cognitive performance, behavioral traits, psychoemotional profiles, and metabolic regulation.

### 4.2. Phenotypes as Multimodal Signatures of Healthy Aging

The combination of these latent factors enabled the delineation of five reproducible aging phenotypes, distinguished by distinct biochemical, functional, behavioral, and psychosocial profiles. Crucially, these phenotypes were independent of both age and sex, aligning with the theoretical framework of multidimensional aging [5,6]. This finding reinforces the concept that inter-individual disparities in health and physiological resilience are driven more by variation in underlying biological systems than by demographic factors. Furthermore, the data illustrate that each phenotype constitutes a stable configuration of correlated changes across multiple systems, rather than an isolated deviation along a single physiological axis. This observation is consistent with the prevailing understanding of aging as a multisystem process of gradual network deterioration [8,11].

A particularly significant finding is the alignment of the objectively defined phenotypes with key subjective indicators, including self-rated health, subjective age perception, and cognitive-emotional scores. This convergence between multimodal biomarker patterns and self-reported experience corresponds to contemporary models of subjective aging [25], which posit that an individual’s perception of their own aging is an integral and clinically relevant component of their biopsychosocial profile.

Within the context of our findings, this suggests that subjective feelings of well-being or vulnerability are not merely psychological constructs but mirror underlying biological states, including inflammatory and stress-related pathways, and may serve as sensitive early indicators of declining physiological resilience. Consequently, the derived phenotypes represent an integrated structure of inter-individual variation, synthesizing objective biomarkers with subjective perceptions. This holistic framework is highly relevant for refining biological age estimations and advancing the goals of preventive and personalized medicine.

The five phenotypes delineated here can also be positioned against the existing landscape of data-driven aging typologies. The “Balanced” phenotype, located closest to the global centroid of the factor space and characterized by preserved cognitive and functional performance despite mild astheno-inflammatory shifts, parallels the normative-aging and “Super-Senior” profiles described by Halaschek-Wiener et al. [9], in which advanced age coexists with the absence of major age-related disease. The “Metabolically Decompensated” phenotype, defined by concurrent insulin resistance, abdominal obesity, systemic low-grade inflammation, and early vascular-aging biomarkers, mirrors the inflammatory-metabolic cluster identified by Valenzuela et al. [7] through network analysis of integrated physiological and psychological profiles, and overlaps with the metabolically adverse longevity subgroup reported by Marron et al. [8] in long-lived families. The “Overloaded” phenotype, in which a favorable biochemical fingerprint coexists with subjective stress and poor sleep, does not map cleanly onto previously described clusters and may represent a population-specific configuration that warrants replication in external cohorts. Finally, the “Anemic” and “Metabolically Subcompensated” phenotypes both fall outside the classical adverse-versus-healthy dichotomy captured in earlier studies, consistent with the hypothesis that integrative, unsupervised approaches can reveal phenotypic configurations masked by disease-centric case/control designs. Taken together, this partial correspondence with prior work supports the biological plausibility of our partition. It demonstrates that the RUSS-AGE cohort captures axes of variation not fully covered by existing Western typologies.

### 4.3. Machine-Learning Validation of Phenotypic Structure

To validate the phenotypes using an independent methodological approach, we developed a supervised multiclass classifier. This model predicted phenotypic membership based solely on the original, unprocessed laboratory, anthropometric, and questionnaire variables. As detailed in Section 3.5, the XGBoost-based classifier demonstrated strong performance in discriminating the five phenotypes (macro-averaged F1-score ≈ 0.75; One-vs-Rest ROC-AUC ≈ 0.93). Critically, performance metrics showed negligible degradation on the held-out test set, confirming the internal reconstructability of the phenotypic partition rather than external generalization to independent cohorts; the latter remains an open task for future multi-center studies.

The greatest confusion arises between Phenotypes 3 and 4 due to their partial overlap in metabolic and functional characteristics, whereas the anemic (P1) and balanced (P5) phenotypes are recognized particularly well. Replicating these findings with CatBoost, a gradient boosting framework with a distinct internal architecture, provides robust evidence that the phenotypic structure is algorithm-independent.

### 4.4. Strengths and Novelty of the Approach

Two-stage analytical design rationale. The MOFA2 latent factorization (with K = 16 factors) and the K-means consensus clustering (K = 5 phenotypes) were deliberately decoupled because the two steps optimize different criteria: MOFA K is selected by the proportion of cross-modality variance captured (EV_total) and is a property of the latent structure, whereas clustering K is selected by partition-stability metrics (within-cluster compactness, between-cluster separation, PAC, ARI across bootstraps). A single-step design that forced K_MOFA = K_phen = 5 would underfit the latent space (EV_total drops from 21.3% at K = 16 to ~11% at K = 8 in our sensitivity analysis; see Supplementary Table S3) and thereby collapse biologically distinct axes—for example, anemia/iron metabolism and inflammation/NT-proBNP—before the clustering algorithm has access to them. The two-stage procedure adopted here is consistent with the recommendations of the original MOFA/MOFA+ methodology papers [10,18].

This study has several key methodological strengths:

1. Multimodal Data Integration: The integration of laboratory, anthropometric, behavioral, and psychosocial data modalities provided a holistic view of inter-individual variability, moving beyond single-system analyses.
2. Robust Handling of Missing Data: The use of the probabilistic MOFA2 framework allowed us to incorporate all available data without imputation, leveraging the inherent structure of missingness to derive more reliable latent factors.
3. Accounting for Demographic Confounders: The statistical removal (residualization) of variance associated with age and sex during preprocessing enabled the isolation of biological and behavioral axes of variation independent of these major demographic factors.
4. Robustness and Independent Validation: The phenotypes demonstrated stability and were validated using an independent supervised machine-learning approach that successfully recapitulated the clusters from the original feature space.
5. Clinically Informed Interpretation: The development and application of a structured clinical variable dictionary ensured that the computational findings were grounded in and interpretable within a biomedical and clinical context.

### 4.5. Limitations and Scope of Interpretation

At the same time, the study has several limitations: the single-center design requires subsequent external validation on independent samples to confirm the universality of the identified phenotypes; the absence of metabolomics, proteomics, and microbiome data limits the biological depth of integration and the accuracy of mechanistic interpretations; the cross-sectional design does not allow for the assessment of transitions between phenotypes and the rates of biological aging at the individual level. Furthermore, varying data completeness across modalities and heterogeneity of coverage remain sources of methodological constraints.

Finally, it is important to emphasize that the predictive modeling component of this study was conducted as a pilot investigation. The XGBoost and CatBoost classifiers were implemented to test the structural stability and algorithm-agnostic nature of the phenotypes, rather than as a final, optimized tool for clinical deployment. Consequently, they were trained without exhaustive hyperparameter optimization. The development of a clinically applicable predictive system would require larger, independent cohorts for external validation, rigorous feature engineering, and careful model calibration.

### 4.6. Prospects for Future Research

This work opens several complementary avenues for future research. First, integrating additional molecular data layers—such as metabolomics, proteomics, and gut microbiome profiling—would substantially enhance the biological resolution of the model. This would allow for a more precise characterization of the molecular mechanisms underpinning each phenotype and could reveal novel biomarkers and therapeutic targets.

Second, longitudinal dynamic phenotyping is a critical future direction. Tracking the same individuals over time to observe stability and transitions between the identified phenotypes would transform this framework from a static classifier into a dynamic model of aging trajectories. This would provide a powerful tool for quantifying individual rates of biological aging and evaluating the efficacy of preventive or therapeutic interventions aimed at modulating these trajectories.

Third, to translate these research findings into practical utility, the development of a web-based application or calculator is warranted. Such a tool could enable the semi-automatic calculation of an individual’s phenotypic profile and corresponding probabilities from a set of input variables. This would facilitate the generation of interpretable, personalized feedback for both individuals and clinicians, supporting preventive health strategies.

Finally, to enable future integration into clinical workflows, the methodology and data formats must be aligned with healthcare information system standards. This would ensure compatibility with electronic health records (EHRs) and pave the way for the potential application of these aging phenotypes in the routine practice of preventive and personalized medicine.

In parallel with these prospective extensions, a dedicated English-language companion manuscript is in preparation that will provide a comprehensive clinical and epidemiological description of the RUSS-AGE cohort, including detailed inclusion/exclusion criteria, the full battery of questionnaire instruments, anthropometric and functional assessments, and laboratory protocols. That paper will serve as the canonical reference for the cohort and will allow subsequent users of the dataset to assess the generalizability of the phenotypes presented here to other Russian-speaking populations.

## 5. Conclusions

This study presents an integrative approach for phenotyping health status using the multimodal factor model MOFA2, applied to the RUSS AGE cohort. Our analysis identified a robust system of 16 latent factors capturing key axes of inter-system variability. Furthermore, we delineated five biologically coherent phenotypes of healthy aging that are largely independent of chronological age and sex.

Preliminary supervised reconstruction of the phenotype labels using gradient-boosting classifiers (XGBoost and CatBoost) demonstrated that the partition is algorithm-agnostic and internally recapitulable from the original clinical, laboratory, and survey variables; this supervised step should be interpreted as an internal consistency check rather than as out-of-sample predictive validation. These findings underscore the potential of data-driven phenotypic stratification for advancing research into the biology of aging. They provide a proof-of-concept foundation on which applied risk-assessment and biological-age tools can subsequently be developed, contingent on longitudinal follow-up and external validation in independent cohorts.

## Data Availability

The data that support the findings of this study, including the trained MOFA2 model and the per-participant phenotype assignments, are available from the corresponding author on request, subject to approval by the Ethics Committee of the Russian Gerontology Clinical Research Center and to compliance with the informed-consent terms signed by RUSS-AGE participants. Public open-access deposition of individual-level data is not permitted under the current consent and ethics framework. Aggregate Supplementary Tables S1–S4 and Supplementary Figures S1 and S2 accompanying this manuscript are openly available as supporting information published with the article.

## Supplementary Materials

The following supporting information can be downloaded at https://www.mdpi.com/article/10.3390/biomedicines14051158/s1. Table S1: Complete dictionary of the 118 variables used in the MOFA2 model (modality, domain, units, likelihood, missing-data proportion, variable-level variance explained); Table S2: Factor loadings 118 × 16 with top-15 positive/negative loadings per factor and per-factor semantic themes; Table S3: Cluster-stability and MOFA-K sensitivity grids (K = 4–9 clusters; K = 8–16 MOFA factors) plus factor-subset sensitivity; Table S4: Detailed per-phenotype characterization (sample composition, top biomarkers with Cliff’s delta, behavioral and clinical profiles); Figure S1: Age-by-sex pyramid of the analytical cohort (10-year strata); Figure S2: Factor-age correlations and factor-sex effects before and after demographic residualization.
