# Supplementary material for: Multimodal Factor Analysis Reveals Five Robust Phenotypes of Healthy Aging in a Russian Population Cohort
Source: Biomedicines. 2026 May 20;14(5):1158. doi: 10.3390/biomedicines14051158 (PMC13204055; doi:10.3390/biomedicines14051158)
Supplement: Supplementary file 1 [file biomedicines-14-01158-s001.zip › Supplementary_Figure_S1_Age_Sex_Pyramid.pdf]

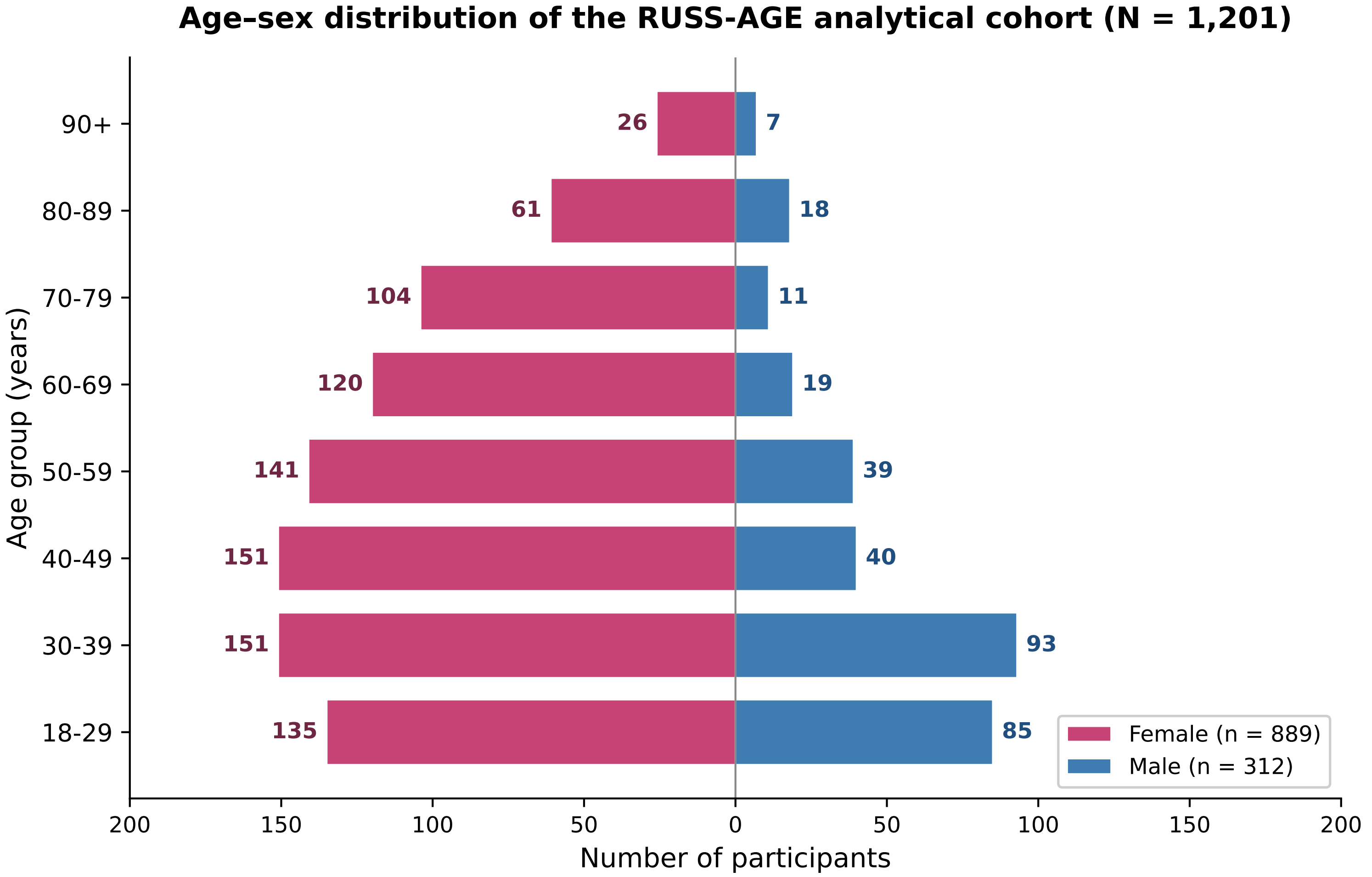

Women: median 50 [IQR 36-67] | Men: median 36 [IQR 29-53]
Overall age range: 18-99 y | Mean ± SD: 49.3 ± 19.6 y
