# Supplementary material for: Multimodal Factor Analysis Reveals Five Robust Phenotypes of Healthy Aging in a Russian Population Cohort
Source: Biomedicines. 2026 May 20;14(5):1158. doi: 10.3390/biomedicines14051158 (PMC13204055; doi:10.3390/biomedicines14051158)
Supplement: Supplementary file 1 [file biomedicines-14-01158-s001.zip › Supplementary_Figure_S2_Residualization_effect.pdf]

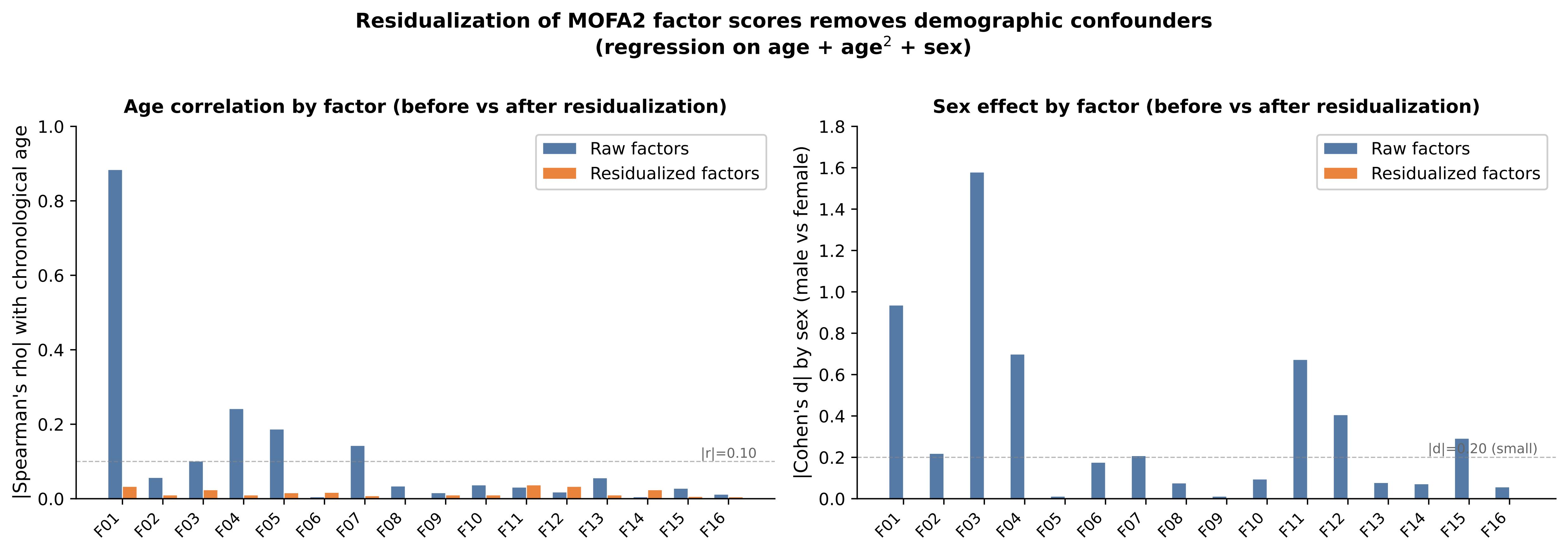

Residualization of MOFA2 factor scores removes demographic confounders
(regression on age + age^2 + sex)
Age correlation by factor (before vs after residualization)
|Spearman's rho| with chronological age
Raw factors
Residualized factors
|r|=0.10
F01
F02
F03
F04
F05
F06
F07
F08
F09
F10
F11
F12
F13
F14
F15
F16
Sex effect by factor (before vs after residualization)
|Cohen's d| by sex (male vs female)
Raw factors
Residualized factors
|d|=0.20 (small)
